# A hidden lung cancer in a patient with granulomatosis with polyangiitis

**DOI:** 10.1259/bjrcr.20190114

**Published:** 2020-09-29

**Authors:** Cheng Xie, Catriona Stoddart, James Bewes, Heiko Peschl, Raashid Luqmani, Rachel Benamore

**Affiliations:** 1 Department of Radiology, Churchill Hospital, Oxford University Hospital Trust, Old Road, Headington, Oxford, OX3 7LE, United Kingdom; 2 Department of Rheumatology, Nuffield Orthopaedic Centre, Oxford University Hospital Foundation Trust, Windmill Road, Headington, Oxford, OX3 7HE, United Kingdom

## Abstract

Granulomatosis with polyangiitis is a systemic necrotizing vasculitis that affects the small- and medium-sized blood vessels. The diagnosis can be challenging since the clinical and imaging findings have similarities with infection, and malignancy. Serologic and histopathological investigations often help confirm the diagnosis. However, this can be falsely reassuring. We present a unique case of the coexistence of vasculitis and squamous cell carcinoma in the same cavitating lung mass. The case highlights the importance of recognizing changes in disease behaviour early to allow for timely management.

## Clinical presentation

**Figure 1.**
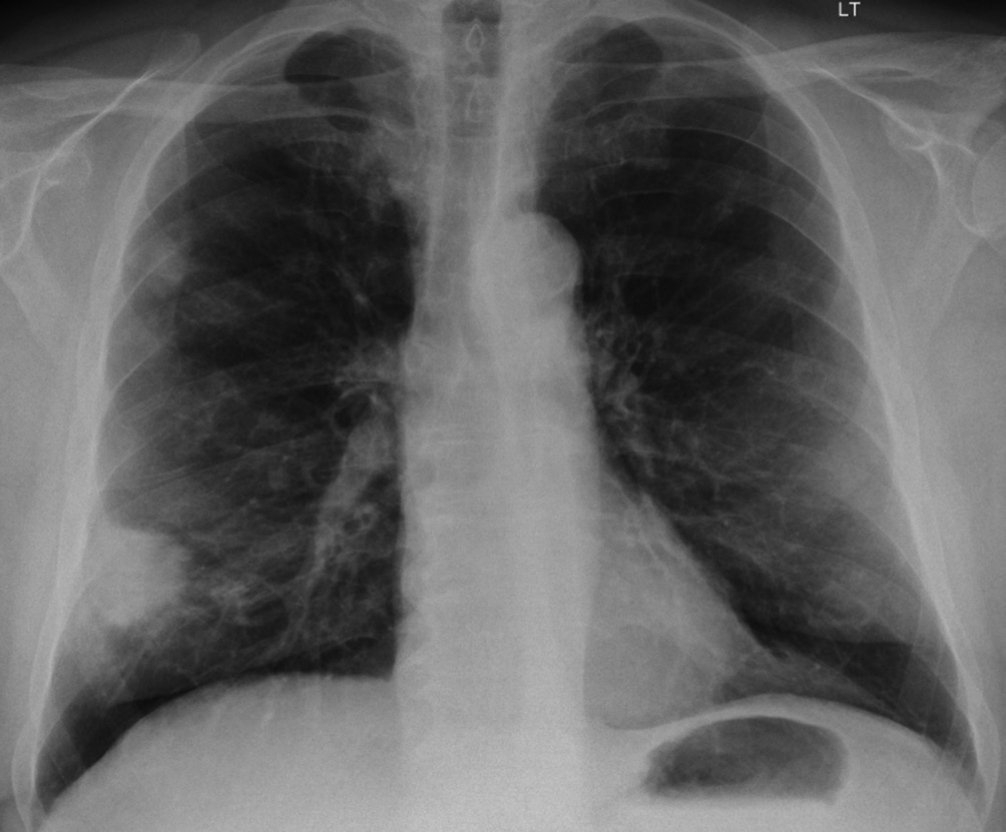
78-year-old male urgently referred by his GP with a chest X-ray showing a spiculated 3.5 cm mass in the right lower zone.

A 78-year-old male was urgently referred by the General Practitioner with a 4 week history of productive cough, occasional haemoptysis and epistaxis with an abnormal chest X-ray showing a spiculated mass in the right lower zone (Figure 1). He had hypertension, and a previous history of treated tuberculosis infection. He was an ex-cigarette smoker. There were no significant findings on clinical examination.

### Investigations

**Figure 2.**
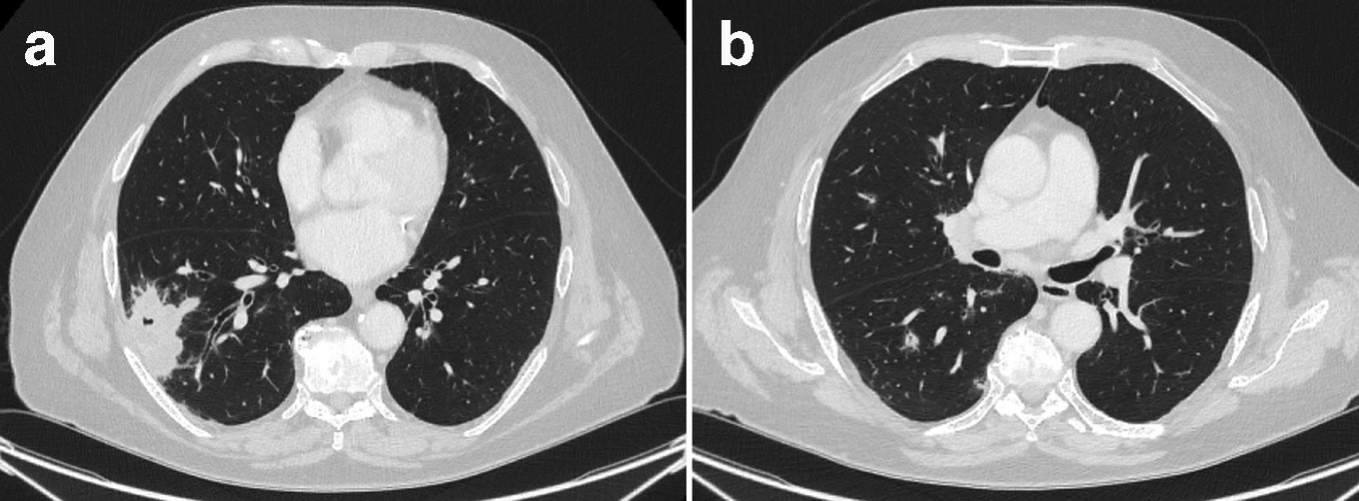
CT showed a lobulated, cavitating 5 cm mass in the right lower lobe (Figure 2a) with bilateral small cavitating nodules (2b).

**Figure 3.**
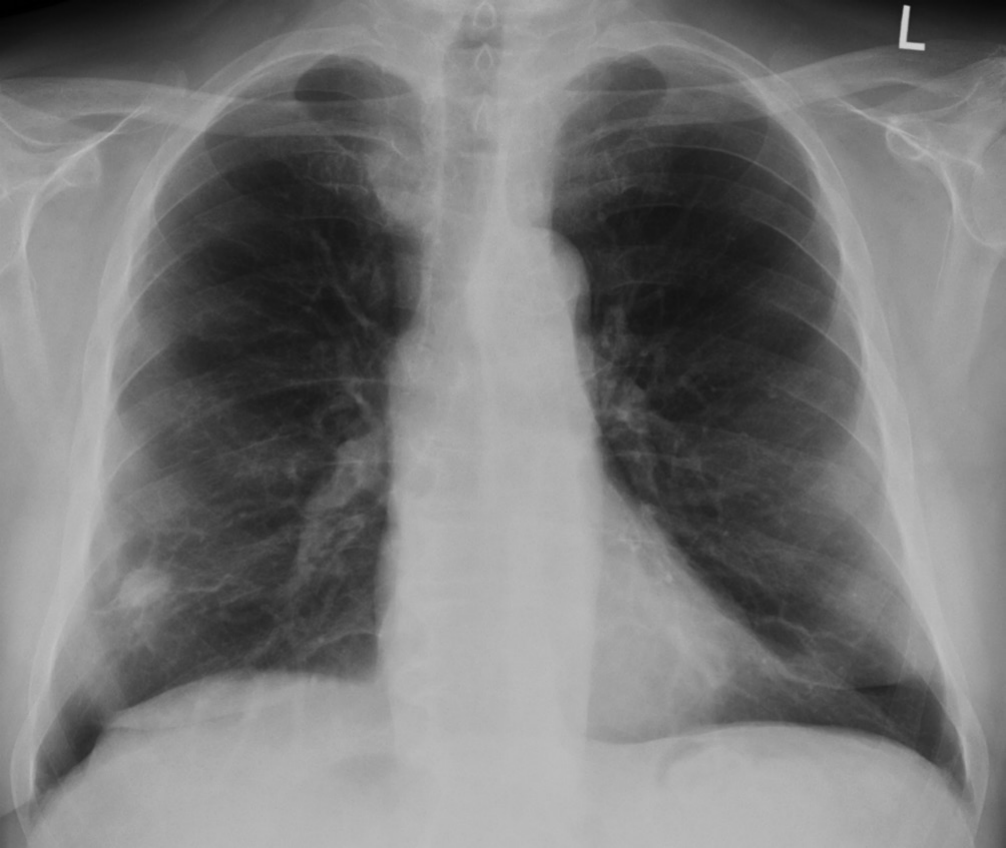
Good treatment response after the first 3 months treatment with follow-up chest X-ray showing a reduction in size of the right lower lobe nodule (2 cm).

The CT scan of the thorax showed a cavitating mass in the right lower lobe (Figure 2a) with bilateral small cavitating nodules (Figure 2b). The patient had antineutrophil cytoplasm antibody (cytoplasmic pattern, cANCA) at a titre of 1/20 and a PR3 level of 14.6 IU ml^−1^. The histopathology from the subsequent CT-guided biopsy of a representative sample of the right lower lobe mass revealed necrotizing granulomatous inflammation, but no evidence of malignancy. He was commenced on subcutaneous methotrexate with a good response both clinically and on imaging (Figure 3). After 3 months, the patient developed renal impairment, which was suspected to be due to a combination of disease progression and/or methotrexate nephrotoxicity. His treatment was switched to high dose intravenous pulses of cyclophosphamide and a tapering dose of oral prednisolone, which is shown to be effective in achieving remission in over 88% of patients within 9 months,^1^ and in accordance with European League Against Rheumatism recommendations.^2^

**Figure 4.**
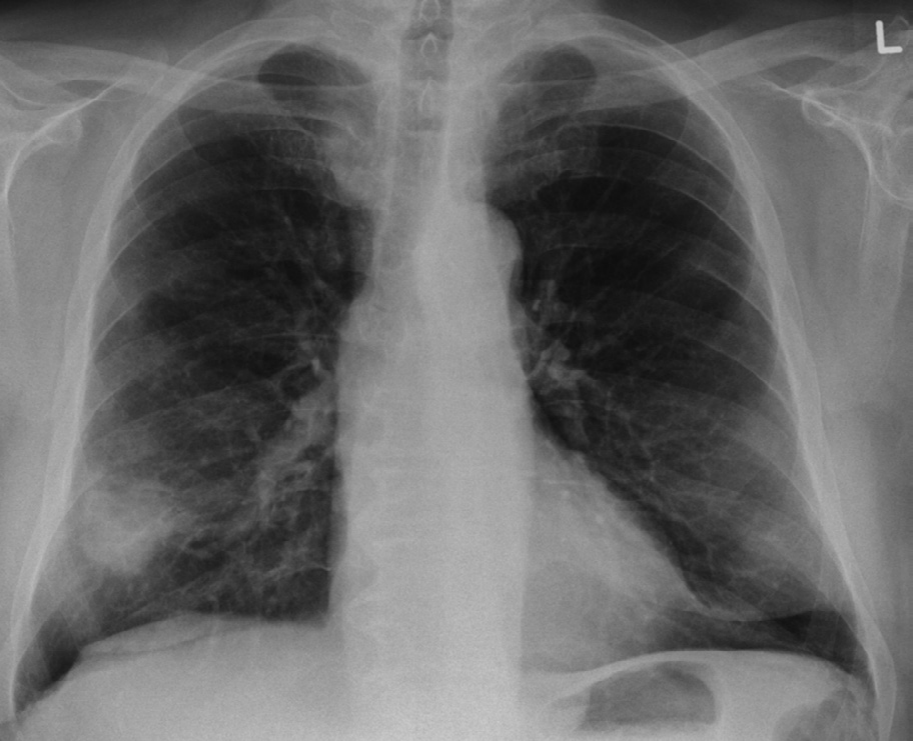
After 3 months, the patient developed a new cough and the chest X-ray showed increase in size of the right lower nodule (4 cm).

**Figure 5.**
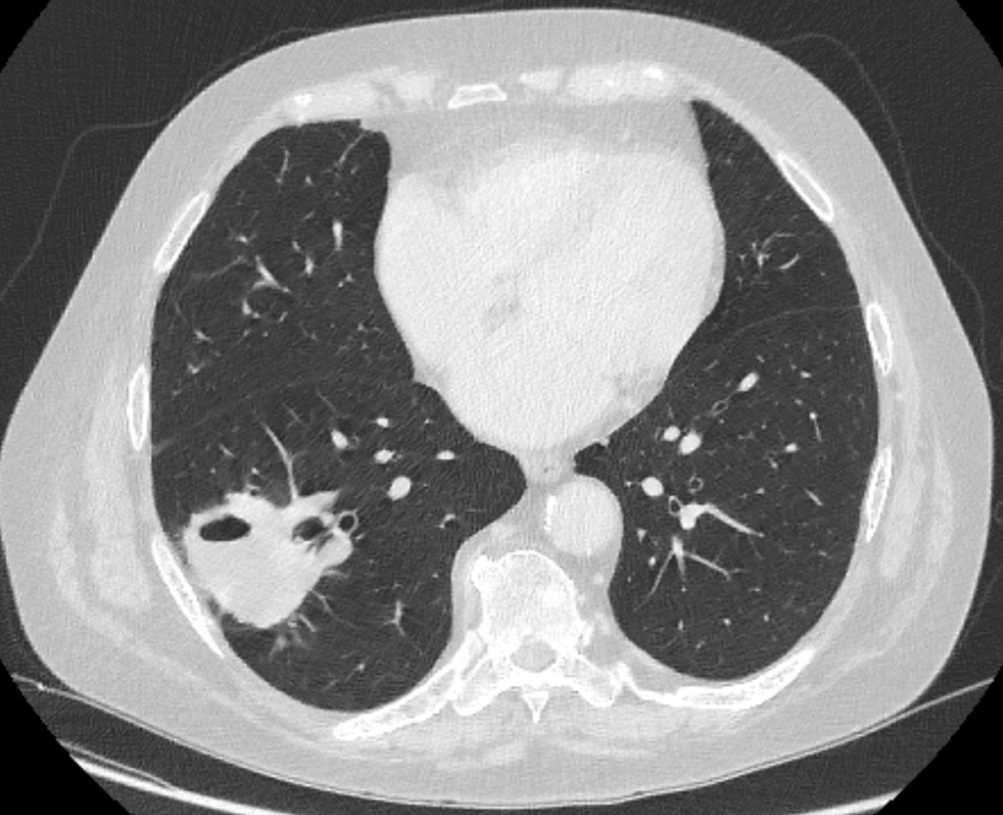
The CT performed at the time of the new cough showed an increase in size of the 2 cm nodule in the right lower lobe into a 5 cm mass with cavitation.

**Figure 6.**
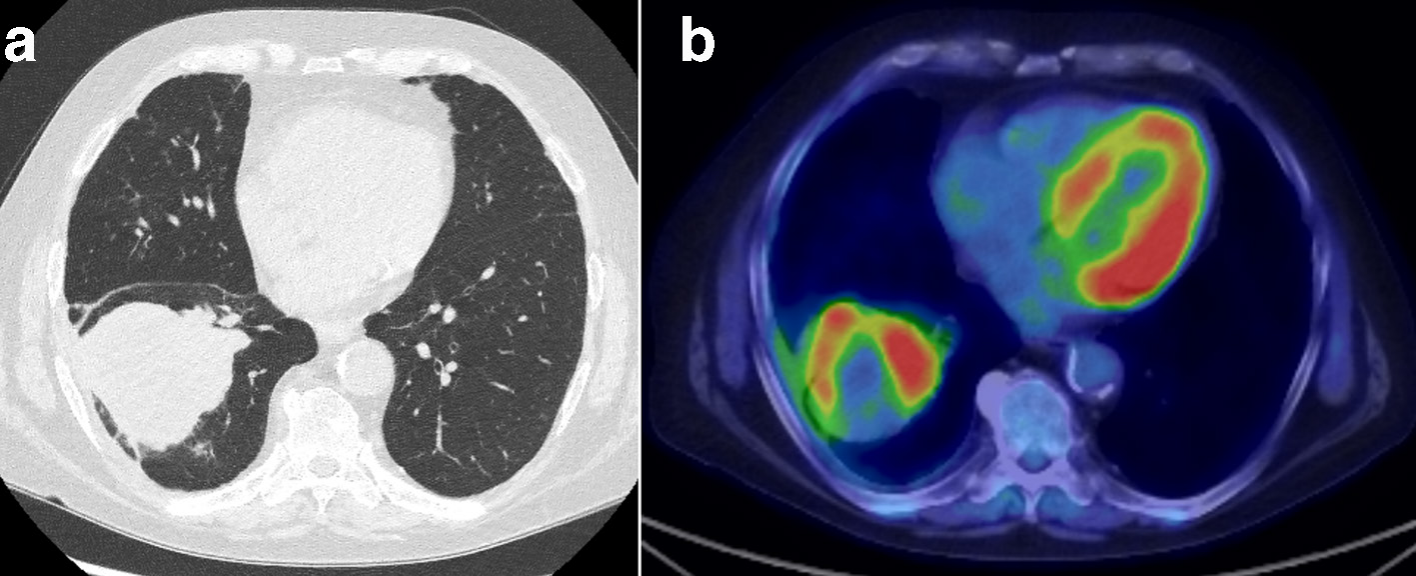
After failure of different combinations of drugs targeting the suspected vasculitis, a repeat CT (6a) enlarging lobulated 7.5 cm mass in the right lower lobe with adjacent pleural involvement; and the PET/CT (6b) showed peripheral FDG avidity and central necrosis.

After 3 months of cyclophosphamide treatment, the patient developed a new cough and his chest X-ray showed an increase in size of the right lower nodule (Figure 4). The clinical impression at this stage was a flare of the vasculitis. The CT confirmed a cavitating mass (Figure 5) at the same location as the initial CT. Over the next 6 months, the patient was treated with mycophenolate mofetil, rituximab, and further cyclophosphamide infusions, on the assumption that he had more resistant vasculitis. However, there was no improvement in his symptoms and he continued to lose weight. This raised the suspicion of an alternative cause and prompted another CT. The CT showed an enlarging lobulated mass at the same location in the right lower lobe with adjacent pleural involvement (Figure 6a). The corresponding PET-CT imaging demonstrated high avidity and central necrosis with right hilar lymphadenopathy (Figure 6b). A repeat biopsy of the mass revealed squamous cell carcinoma (SCC).

### Differential diagnosis

The working diagnoses on presentation after the initial CT (Figure 2) were infection (including tuberculosis and aspergillosis), vasculitis and lung malignancy. Cavitating lesions, particularly in a peripheral and lower zone distribution are typical of vasculitis, but can also be caused by tuberculosis or SCC. Therefore, biopsy was required and in conjunction with a positive ANCA, confirmed the diagnosis of granulomatosis with polyangiitis (GPA). After an initial apparent period of treatment response (3 months), he worsened, suggesting that his vasculitis was resistant and required a change in therapy. There were no indications to suggest an intercurrent infection. However, the inadequate disease control following 6 months of therapy with different immunosuppressant agents (including Rituximab) raised the concern that there might be another underlying cause.

### Outcome

Following confirmation of SCC, the patient’s PET/CT confirmed a disease staging of T4N2M0. He was urgently referred to the lung cancer multidisciplinary team for further management.

## Discussion

GPA is a multisystem disorder of necrotizing vasculitis involving small and medium sized blood vessels and is typically found to be associated with the presence of ANCA. Pulmonary nodules with cavitation are the main radiological feature of GPA affecting the lungs. Lymphadenopathy is rare.^3^ Given the imaging similarities between vasculitis and malignancy, it has been well documented that vasculitis could mimic malignant tumours and infection (tuberculosis, aspergillosis), which can make the diagnosis challenging.^4–7^ There is also evidence that long-term treatment of vasculitis including the use of cyclophosphamide increases subsequent lung cancer risk.^8–11^ A case series reports the development of lung cancer within 2 years of treatment.^12^

The patient in this case had positive serology and histopathology for GPA and responded well to treatment in the first 3 months. In retrospect, the short period of response to treatment before the malignancy progressed suggests the possibility of a coexistent malignant tumour at the same site as the vasculitis, rather than either resistant vasculitis or therapy-induced lung cancer. We suspect that the vasculitic component of the right lower lobe mass initially responded to therapy, and the residual nodule at the end of the 3 months treatment period (Figure 3) was the malignant component, which continued to grow despite different combinations of drugs targeting the vasculitis. An early CT was not performed because of initial improvement of the lung mass and other nodules. However, once a radiograph showed interval increase in size of the remaining nodule, reassessment of this with a repeat CT was immediately recommended and done. We did not feel a contrast-enhanced CT would add any additional value since both vasculitic and malignant tumour would demonstrate contrast enhancement. To our knowledge, there have been one previous case report of a possible GPA coexistence with lung cancer.^13^ Although rare, our case highlights that we must remain vigilant in suspecting other potential causes, such as infection or malignancy, to explain an apparent early relapse or failure to respond to conventional immunosuppressive therapy even in patients who have a confirmed diagnosis of vasculitis.

## Learning points

1. Vasculitis could mimic malignant lung tumour and vice versa, and even co-exist at presentation.
2. Clinicians and radiologists should remain vigilant of any abnormal behaviour of the disease, even after serology and histopathology confirmation.
3. Early detection of any change in the disease behaviour should trigger discussion of the case at a multidisciplinary setting for further management.
